# Impact of the Canadian CT head rule supplemented by the original published minimum inclusion criteria to assist emergency department clinicians’ assessment of patients presenting post fall from residential aged care: a retrospective audit

**DOI:** 10.1186/s12877-022-03284-0

**Published:** 2022-07-21

**Authors:** Charlene Lee, Jonathan Beavers, Jonathan Pham, Liam Hackett, Joseph Miller, Paul Buntine

**Affiliations:** 1 Department of Geriatric Medicine, Eastern Health, Melbourne, Australia; 2 Eastern Health Clinical School, Monash University, Melbourne, Australia; 3 Department of General Medicine, Eastern Health, Melbourne, Australia; 4 Box Hill Hospital Emergency Department, 5 Arnold Street, Box Hill, Victoria, 3128 Australia

**Keywords:** Computed tomography, Brain, Emergency, Decision support techniques, Clinical score, Residential aged care, Canadian CT head rule, Geriatric, Falls, Head injury, Intracranial haemorrhage

## Abstract

**Background:**

A large number of CT brain (CTB) scans are ordered in the ED for older patients with a confirmed or possible head strike but no ongoing symptoms of a head injury. This study aimed to evaluate the effect of the Canadian CT head rule supplemented by the original published minimum inclusion criteria to assist clinician assessment of the need for CTB following minimal trauma fall in patients presenting from residential aged care facilities to a major metropolitan emergency department (ED).

**Methods:**

This study was conducted as a pre- and post-intervention retrospective audit. The intervention involved implementation of a decision support tool to help clinicians assess patients presenting to the ED following a fall. The tool integrated the Canadian CT Head Rule (CCHR) in conjunction with a simplified set of inclusion criteria to help clinicians define a minimum threshold for a “minor head injury”. Outcome data pertaining to CT brain ordering practices and results were compared over symmetrical 3-month time periods pre- and post-intervention in 2 consecutive years.

**Results:**

The study included 233 patients in the pre-intervention arm and 241 in the post-intervention arm. Baseline demographics and clinical characteristics were similar in both groups. There was a 20% reduction in the total number of CTB scans ordered following tool implementation, with 134 (57.0%) scans in the pre-intervention group and 90 (37.3%) in the post-intervention group (*p* <  0.01). The diagnostic yield in the pre- and post-intervention groups was 3.7 and 5.6% respectively (*p* = 0.52). No variation was observed in medical management between groups, and no patients in either group underwent neurosurgical intervention.

**Conclusions:**

Use of the CCHR supplemented by the original published minimum inclusion criteria appeared to safely reduce the number of CTB scans performed in residential aged care facility residents presenting to an ED after a fall, with no associated adverse outcomes. A larger study across multiple centres is required to determine widespread efficacy and safety of this tool.

## Introduction

Falls in older people are a common and serious emergency department (ED) presentation associated with significant morbidity and mortality. In this population subgroup (aged 65 years and older), head injury is the most common single cause of mortality in those individuals who fall [1, 2]. In Australia, an estimated 75% of all injury-related hospitalisations in older people can be attributed to a fall, and of these cases, approximately 25% obtained a head injury, a rate that has doubled between 2007 and 2017 [3]. When compared with their community-dwelling counterparts, residents of residential aged care facilities are particularly at risk of fall-related injury in the context of a greater prevalence of frailty and physical and cognitive comorbidity in this population. In addition to the substantial personal cost that a fall can have upon an individual’s health, falls are also associated with substantial growing financial costs to society and healthcare systems. An estimated 3.9 billion dollars is spent across Australia each year on fall-related health expenditure [3].

Well-established clinical decision rules exist to guide computed tomography (CT) recommendations in patients presenting to EDs with minor head injury; these include the most widely used Canadian CT Head Rule (CCHR). Clinicians have often interpreted this rule as suggesting that imaging should be performed on all patients aged ≥65 years with a proven or possible head strike, regardless of their clinical presentation or pre-morbid state and without considering the original inclusion criteria for application of the rule. In addition to being largely over the age of 65 (a “high risk” criteria in the CCHR), residents in residential aged care facility settings often possess pre-existing physical or cognitive frailties, which can frequently lead to difficulty in establishing cognitive baseline or interpreting Glasgow Coma Scale (GCS) scores. This has resulted in widespread, routine CT brain scanning in older patients presenting from residential aged care facilities, even when detailed history taking suggests a trivial injury and no associated symptoms. Additionally, if an intracranial haemorrhage (ICH) is diagnosed, a conservative approach to care may often be more appropriate within this group of older, frailer patients, and the possibility of therapeutic harm from factors such as prolonged ED stay or a need for intra-procedure sedation is rarely considered.

We hypothesised that the CCHR, supplemented by minimum inclusion criteria, would safely reduce the need for CT brain (CTB) scans in patients from residential aged care facilities presenting to ED following a fall.

## Methods

Study Design.

**Fig. 1:**
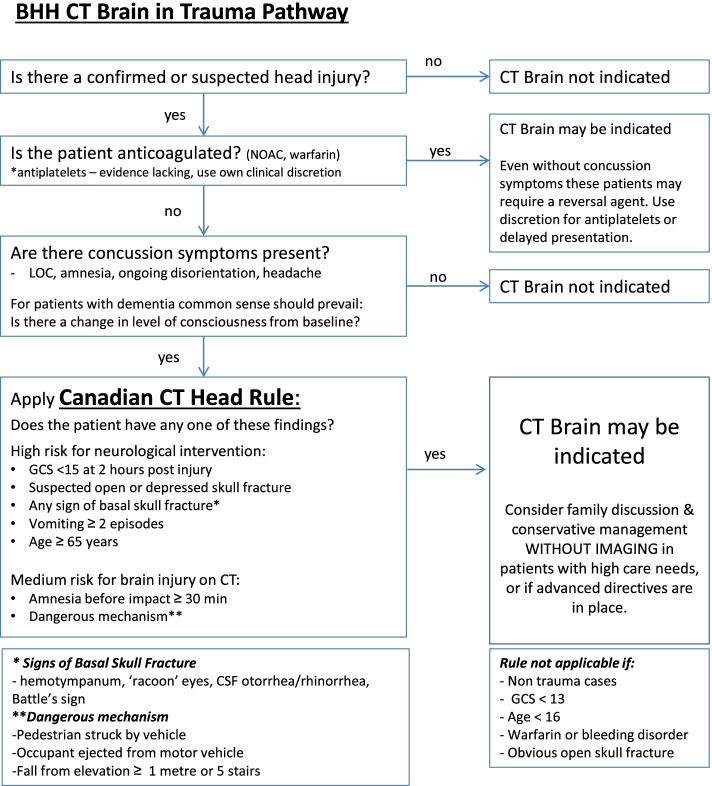
Diagnostic support tool implemented in the Emergency Department

Box Hill Hospital is a major metropolitan hospital in Melbourne, Australia with an annual ED census of approximately 71,000. Prior to the intervention, clinicians ordered CTB scans for patients aged > 65 with possible head strikes at their own discretion. While the CCHR may have been followed on occasion, this was undocumented, and clinicians lacked a thorough understanding of its inclusion criteria. As part of a wider initiative to optimise diagnostic test ordering within the ED, a decision support tool was implemented with an aim to safely rationalise CTB ordering practices in the work up of patients presenting with a minor head injury (Fig. 1).

We conducted a pre- and post-intervention retrospective audit reviewing the outcomes associated with the implementation of a paper-based decision support tool at the Box Hill Hospital ED from the 1st of April 2019 in patients presenting from residential aged care facilities following a fall. ED clinicians were provided information regarding use of the tool via daily morning meetings and emails and were encouraged to apply the tool to all patients with a possible head strike. Radiographers triaging imaging referrals were advised only to proceed with a CTB on receiving a completed copy of the tool. Review and comparison of outcome data for a 3-month convenience sampling of symmetrical time periods from May 1st to July 31st in 2018 and 2019 was completed. To allow for 1 month’s embedding of the tool prior to audit data collection, data from April were not included. Audit approval (reference QA19/091) was obtained from the Eastern Health Human Research Ethics Committee (EHHREC).

### Intervention

This decision support tool (Fig. 1) was based on the CCHR, which is a highly sensitive tool derived from the identification of five high-risk factors (“failure to reach a Glasgow Coma Scale (GCS) score of 15 within 2 hours, suspected open skull fracture, any sign of basal skull fracture, vomiting ≥2 episodes or age ≥65 years”) as well as two medium-risk factors (“amnesia before impact >30min and dangerous mechanism of injury”). The definition of “minor head injury” used when deriving this rule included “a history of loss of consciousness (LOC), amnesia or confusion as well as a GCS score of at least 13–15” [4]. Our tool incorporated a simplified set of inclusion criteria to help clinicians define a minimum threshold for “minor head injury” before application of the CCHR, whilst reminding them to be particularly wary of patients taking anticoagulants. The tool also encouraged family discussion and careful consideration of conservative management without imaging in patients for whom the risks of scanning were likely to outweigh the benefits.

### Study population

All patients who presented to the ED from a residential aged care facility following a fall during the aforementioned inclusion dates (regardless of GCS score) were included in this study. Patients were identified from the Victorian Emergency Minimum Dataset (VEMD) and filtered to include only those with “residential aged care facility” listed as their place of residence. Subsequent manual review of the electronic medical record (EMR) by a single researcher confirmed those who presented with a fall-related issue. Patients who were either identified as having been incorrectly assigned with “residential aged care facility” as their place of residence or who were only a temporary resident (such as respite accommodation) were excluded from the study.

### Data collection

Demographic data, including patients’ dates of birth and gender, were collected from the VEMD. Clinical data such as fall presentation characteristics, anticoagulation, and cognitive impairment history were manually extracted from the EMR. Fall presentation characteristics identified included whether the fall was witnessed, whether there was associated head strike, head injury or concussive symptoms, as well as any focal neurological deficit or alteration to baseline GCS. Total number of medications was recorded as a surrogate comorbidity measure. This has been demonstrated elsewhere to have predictive value for mortality, future physician visits and health expenditure [5]. Calculated Falls Risk Assessment Tool (FRAT) scores completed by nursing staff in the ED were also collated. The FRAT is a validated, reliable and easy-to-use screening tool that has been shown to have moderately predictive value for future falls in older adults [6].

Outcome measures were similarly ascertained from the EMR. This included frequency and result of CT scans performed in the ED, use of physical or chemical restraint and subsequent medical and surgical management. Diagnoses of intracranial haemorrhage (ICH) were categorised as follows: “intracerebral”, “extradural”, “subdural”, “subarachnoid” or “intraventricular haemorrhage confirmed on CTB.” CTBs subsequently performed on patients who were admitted to the ward from ED were defined as “ward CTBs”.

Each patient’s EMR was reviewed 2 weeks before the index ED presentation to capture any delayed or late ICH presentations from a prior fall presentation that had not resulted in a transfer to ED for assessment as a result of the fall. Patients’ EMRs were also reviewed 2 weeks after the index case to identify any missed ICH presentations who had subsequently represented. Details of prior or subsequent ED or inpatient management, including follow up CTB, were identified. This was limited to interrogation of medical records held within the Eastern Health network; however, it included any correspondence received from external medical providers such as general practitioners or non-Eastern Health hospitals.

### Statistical analysis

Clinical features at baseline and follow-up for each time period are reported as summary statistics. Means and standard deviations are presented for parametric data; medians and interquartile ranges are presented for non-parametric data. Comparisons between time periods were made using Student’s t-test (or Fisher’s exact test, where appropriate) and the Wilcoxon rank-sum test, respectively. *P*-values lower than 0.05 were considered statistically significant. Comparisons of outcomes between the two time periods for patients with ICH are reported as simple numerical values. All analyses were performed using STATA version 13.

## Results

A total of 1600 cases were reviewed, with 474 cases meeting inclusion criteria. There were 233 patients in the pre-intervention group and 241 in the post-intervention group. Baseline demographic and clinical characteristics of the two groups were similar (Table 1). There were similar numbers of patients receiving anticoagulant therapy in each group (108; 46.4% vs 103; 42.7%). A higher proportion of subjects were taking a direct oral anticoagulant (DOAC) post-intervention compared to pre-intervention (13.7% vs 7.7%).

**Table 1:** Baseline characteristics of pre- and post-intervention groups

	2018 (Pre-intervention) ***n*** = 233	2019 (Post-intervention) ***n*** = 241	***p***-value
Age range	89 (85–92)	88 (84–93)	0.02
median (IQR)			
Gender n (%)			0.00
Female	134 (57.5%)	173 (71.8%)	
Male	99 (42.5%)	68 (28.2%)	
Medication total median (IQR)	7 (4–11)	8 (5–11)	0.36
Medication missing n (%)	24 (10.3%)	23 (9.5%)	0.78
Cognitive impairment n (%)	151 (64.8%)	183 (75.9%)	0.01
Mobility prior to fall n (%)			0.02
Non-ambulant Ambulant Not documented	41 (17.6%) 176 (75.5%) 16 (6.9%)	24 (10.0%) 210 (87.1%) 7 (2.9%)	
FRAT risk n (%)			0.00
High risk Not documented	191 (82.0%) 42 (18.0%)	224 (93.0%) 17 (7.0%)	
Anticoagulation n (%)			0.66
Anticoagulation No anticoagulation Not documented	108 (46.4%) 117 (50.2%) 8 (3.4%)	103 (42.7%) 131 (54.4%) 7 (2.9%)	
Type of anticoagulation n (%)			0.00
Antiplatelet DAPT Warfarin DOAC Antiplatelet + warfarin DAPT + DOAC or warfarin	64 (27.5%) 1 (0.4%) 17 (7.3%) 18 (7.7%) 5 (2.1%) 0 (0.0%)	48 (19.9%) 7 (2.9%) 12 (5.0%) 33 (13.7%) 0 (0%) 0 (0%)	
LMWH Antiplatelet + LMWH Antiplatelet + DOAC	0 (0%) 2 (0.9%) 1 (0.4%)	0 (0%) 0 (0%) 3 (1.2%)	
Witnessed fall n (%)			0.13
Witnessed Not documented	21 (9.0%) 15 (6.4%)	26 (10.8%) 27 (11.2%)	
Headstrike* n (%)			0.75
Headstrike Not documented	110 (47.2%) 78 (33.5%)	118 (49.0%) 73 (30.3%)	
Head injury** n (%)			0.02
Head injury Not documented	86 (37.0%) 89 (38.2%)	105 (43.6%) 63 (26.1%)	
Late presentation n (%) ***	1 (0.4%)	0 (0%)	0.49
LOC n (%)			0.33
LOC Not documented	2 (0.9%) 121 (52.0%)	6 (2.5%) 129 (53.5%)	
Headache n (%)			0.87
Headache Not documented	14 (6.0%) 167 (71.7%)	15 (6.2%) 177 (73.4%)	
Vomiting n (%)			0.08
Vomiting Not documented	10 (4.3%) 191 (82.0%)	9 (3.7%) 180 (74.7%)	
Neurological deficits n (%)	4 (1.7%)	5 (2.1%)	0.78
Change in GCS n (%)			0.19
Change in GCS No change in GCS Not documented	23 (9.9%) 160 (68.7%) 50 (21.5%)	23 (9.5%) 149 (61.8%) 69 (28.6%)	
GCS in ED n (%)			0.45
Not documented < 13 ≥13	6 (2.6%) 27 (11.5%) 200 (85.8%)	2 (0.8%) 18 (7.4%) 221 (91.7%)	

*FRAT* Falls Risk Assessment Tool, *DAPT* Dual antiplatelet therapy, *DOAC* Direct oral anticoagulant, *LMWH* Low molecular weight heparin, *LOC* Loss of consciousness, *GCS* Glasgow Coma Score

* ‘Headstrike’: confirmation that the patient actually hit their head when they fell

** ‘Head injury’: documentation of a visible injury (e.g., bruising or a laceration) associated with a headstrike

*** ‘Late presentation’: patients who presented with an intracranial haemorrhage following a time lag from their initial fall injury. This was captured by reviewing 2 weeks of medical records prior to the index presentation to determine if there had been any previous fall injury

**Fig. 2:**
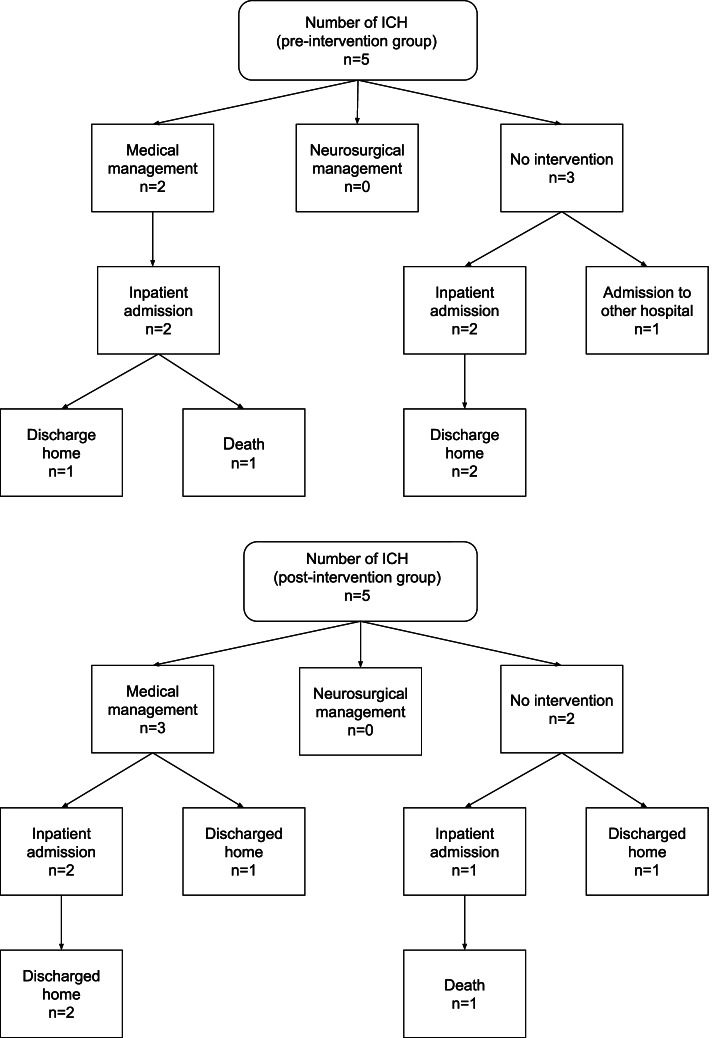
Flow diagrams representing outcomes for diagnosed intracranial haemorrhages in the pre- and post-intervention groups. ***Note*:** ‘Medical management’ refers to pharmacological management, which may include alteration to the patient’s pre-existing medications or prescription of a reversal agent to anticoagulant therapy

**Fig. 3:**
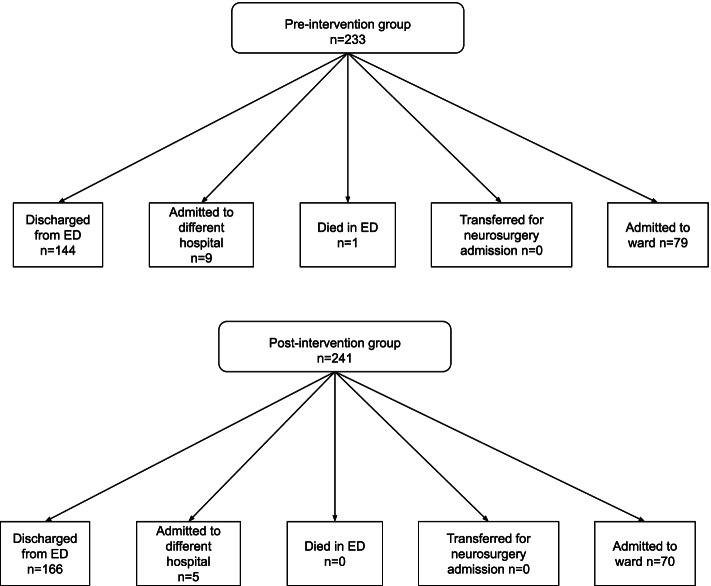
Flow diagrams representing progress of study participants after ED treatment in the pre- and post-intervention groups

A total of 134 (57.0%) CTB were performed in the pre-intervention group compared to 90 (37.3%) in the post-intervention group (difference: − 19.7%, *p* <  0.01). Other clinically significant outcomes were similar following support tool implementation, including the number of cases with ICH (with 5 observed during each time period) and similar rates of medical and surgical management for both groups (Table 2). No statistically significant change was observed in diagnostic yield (calculated as the percentage of diagnosed ICH per number of CTB ordered) between the pre and post intervention groups (3.7% vs 5.6%, *p* = 0.52). No patients in either group underwent neurosurgical intervention. By applying Hanley and Lippman-Hand’s [7] ‘rule of three’, we can conclude with 95% confidence that the frequency of delayed or missed diagnosis of a clinically significant finding requiring neurosurgical intervention did not exceed 1.24% in the post-intervention group. Medical management was defined as withholding or ceasing anticoagulation, anticoagulation reversal or other medication change(s), and was observed with 2 patients in the pre-intervention group (both of whom received anticoagulation reversal) and with 3 patients in the post-intervention group (where 1 patient received anticoagulation reversal, 1 patient had anticoagulation withheld or ceased and 1 patient received a different medication change; Fig. 2). There was a single in-hospital death in each group. The progress of study participants following ED treatment was largely similar (Fig. 3).

**Table 2:** Comparison of pre- and post-intervention outcomes

	2018		2019				
	(*N* = 233)	%	(*N* = 241)	%	Difference		*p*-value
ED CTB performed	134	57.0%	90	37.3%	19.7%		< 0.01**
Confirmed ICH on ED CTB	5 / 134	3.7%	5 / 90	5.6%	1.9%		0.52
Chemical restraint used in ED CTB	4 / 134	3.0%	2 / 90	2.2%	0.8%		1.0
Physical restraint used in ED CTB	0 / 134	0.0%	0 / 90	0.0%	0.0%		–
Missed presentation**	1	0.4%	2	0.8%	−0.4%		1.0
Ward CTB performed	14/79	17.7%	13/69	18.8%	−1.1%		0.86
Abnormal ward CTB	3 / 14	21.4%	3 / 13	23.1%	−2.3%		1.0
ICH on ward CTB	1 / 14	7.1%	2 / 13	15.4%	−8.3%		0.60
Neurosurgical intervention	0 / 134	0.0%	0 / 90	0.0%	0.0%		–

*ED* Emergency Department, *CTB* Computed tomography of the brain, *ICH* Intracranial haemorrhage, Ward CTB = CTBs subsequently performed on patients who were admitted to the ward from ED

** ‘Missed presentation’: patients who represented with an intracranial haemorrhage not diagnosed on the initial fall presentation. This was captured by reviewing two weeks of medical records following the index presentation

In the post-intervention group, there were 2 cases of delayed or missed ICH diagnosis in patients who did not have a CTB in the ED. In the first case, a consensus decision was made with the medical treatment decision maker (MTDM) for a conservative approach to care given the patient’s underlying frailty and comorbidities. However, the decision not to further investigate with imaging was subsequently altered in the context of a further representation to ED several days later with progressive neurological symptoms and following re-discussion with the MTDM. The second patient had a delayed scan after transfer to an external hospital seeking specialist opinion on an unrelated issue. Imaging revealed a chronic subdural haemorrhage of uncertain duration that was managed conservatively.

## Discussion

This study demonstrates that the application of a decision support tool in the ED based on the CCHR coincided with a 20% reduction in the proportion of residential aged care facility residents undergoing CTB after presenting to ED with a fall. A non-significant trend towards increased diagnostic yield was observed. There were no differences in neurosurgical management or other measurable patient outcomes between the pre- and post-intervention groups.

A wide variation has been reported in the diagnostic yield of CT imaging for an acute ICH in older adults following a fall, ranging from 1.6 to 11.6% [8, 9]. A systematic review by de Wit et al. identified a pooled incidence of 5.2% from 11 studies which specifically looked at patients aged 65 years or above who presented to the ED following a fall [10]. Although our study specifically selected only patients from residential aged care facilities (and could potentially represent a more frail cohort at risk for ICH than those reported elsewhere) our findings appear comparable, with yields of 3.7% in the pre-implementation period and 5.6% during the post implementation period, with the latter possibly being attributable to the more targeted scanning strategy as a result of the intervention. A low baseline yield within this sub-population of ED attendees could, however, also be affected by a potentially greater tendency for residential aged care facility residents to be referred to hospital following a fall compared to patients who self-present from the community. This is an important consideration, particularly in our local Australian context, given the Aged Care Royal Commission’s emphasis on falls prevention [11] and a recent public report from the Coroner’s Court in South Australia that encourages CTB scans in any residential aged care facility residents with a possible head strike who are on anticoagulation therapy [12]. It is likely that this directive, questioned in a recent publication by Green et al. [13], has resulted in a reduced threshold for care providers in residential aged care facilities to seek hospital assessment when one of their residents falls. Further research would be required to determine how these factors interrelate.

In our study, no patients in either group underwent neurosurgical intervention. These findings are consistent with similar studies where surgical intervention ranged from 0 to 5% [1, 13, 14]. Comparison of rates of medical management for patients presenting with a fall is more difficult, in part due to the heterogenous nature of possible interventions which can also depend upon other existing comorbidities. For example, anticoagulation cessation can be considered a medical intervention in this context but is of course only a potential intervention in those already taking such therapy. We identified a single study by Sartin et al. [15] that described 29.4% of patients with a confirmed “acute injury” on imaging receiving medical intervention in the form of medication changes; however, this included patients aged 55 years and older with a GCS of 15 on presentation. Fifty percent of patients with a diagnosed ICH in our study received medical intervention, reflecting a much higher rate of intervention that is likely due to our focus on an older, frailer cohort. Cessation of anticoagulation or antiplatelet use was the most common type of medical management seen in our study, yet these medications are less likely to be present in a younger cohort.

A similar recently published quality improvement initiative was performed in Toronto, Canada by Masood at al [16].. During initial root cause analysis, the authors also identified that the CCHR was applied to many patients with “minimal head injury”, rather than those with only “minor head injury”. Following the introduction of a comprehensive education campaign and a “modified Canadian CT Head Rule checklist” to overcome this issue, they observed a reduction in CTB rates in adults of all ages by 13.9% at 3 months and 8% at 16 months, with no increase in adverse outcomes. A point of difference in our study was our much narrower focus on older patients from residential aged care facilities. The greater reduction in CTB scanning rates associated with our tool may be related to the natural tendency of clinicians to err on the side of caution and investigate more frequently with head imaging in this particular cohort, especially in light of the above-mentioned coroner recommendation. As such, CTB rationalisation with tool implementation in this group has potential for a relatively larger reduction. Our consideration in a change from baseline GCS rather than using a threshold of any GCS < 15 in conjunction with incorporation of goals of care discussions may also have contributed to the greater reduction in CTB scanning rates that we observed.

Despite the well-established nature of the CCHR, there has been little exploration of its use explicitly in the older person. To the best of our knowledge, our study is the first to implement the CCHR as an integrated decision support tool specifically to assess the older patient presenting to the ED. Many clinicians continue to interpret the rule as suggesting that all patients aged > 65 require a CTB after a head strike without considering whether it was appropriate to apply the rule in the first place. In a retrospective cohort study, Fournier at al [17]. demonstrated that increasing the high-risk age criteria from 65 years to 75 years was associated with an increase in the sensitivity of the CCHR. This could translate into a 25% reduction of CT scans in those aged 65–74 years without compromising diagnosis of “clinically important brain injuries”. Although their mechanism for reducing CTB in the elderly was different to ours, this further demonstrates that recalibrating clinicians to apply CCHR in a more select cohort, either by increasing the age definition of “high risk” or by tightening applicability criteria within those presenting from residential aged care facilities following a fall appears to have potential to safely reduce CTB utilisation.

Whilst medical investigations can provide useful prognostic information and assistance with treatment decision making [18, 19], the indiscriminate use of low-yield tests has numerous associated healthcare and patient implications. A simple calculation based on an Australian Medicare rebate amount of $230.40 for each CTB [20] and the 20% observed reduction in scanning rate that we observed would equate to a saving of $40,550 per annum in our department, although it is likely that the real figure is much higher when the effect on ED patient flow and follow-up of incidental findings are also considered [21, 22]. *Primum non nocere* (“first, do no harm”); also important is the patient experience and consideration of avoiding unnecessary or burdensome interventions in a vulnerable cohort. Appropriately limiting investigations has potential to improve quality of care provided via such means as avoiding chemical or physical restraints, reduction in ED length of stay, reduction of incidental findings on imaging and potential prevention of cascading investigations or hospital admission that may not be indicated and of little clinical benefit. These potential positive outcomes could also ultimately circumvent exposure to numerous risk factors that may lead to development of delirium in this susceptible cohort and subsequently prevent the significant associated morbidity.

### Limitations

This study followed a retrospective audit design that relied on interpretation of existing electronic documentation and did not allow for detailed matching of the two groups. Accuracy and availability of clinician documentation may influence study results and limitations related to missing data, including that pertaining to patients’ medications and fall characteristics (see Table 1) need to be acknowledged. Incomplete adherence to the pathway during the post-intervention period also needs to be considered, where factors like high frequency of staff rotation, variable levels of junior staff supervision and pre-existing clinical biases may have impacted upon clinician uptake of the pathway. In addition, our simple comparison of means between pre- and post-intervention groups does not account for possible pre-existing trends, changes in clinicians’ approaches to falls management or changes in clinicians’ CTB ordering practices. Nevertheless, our use of symmetrical time periods (12 months apart at the same institution) reduces the potential impact of such confounders.

Follow-up of patients with regard to missed pathology or delayed diagnosis was limited to Eastern Health hospitals or received correspondence and so may not capture all individuals who subsequently presented to other health networks or who may have subsequently died in the community. However, it is unlikely that a patient from a residential aged care facility would represent to a different health service in such a circumstance, or that Eastern Health would not be notified of such an incident.

Finally, our use of data from a single centre may limit the external applicability of these findings.

## Conclusion

Among older people, falls are a growing global health problem associated with risk of serious adverse sequelae and increasing healthcare costs. This simple retrospective audit suggests that the addition of minimum inclusion criteria to the CCHR can safely reduce investigation with CTB in older patients presenting to the ED from residential aged care facilities following a fall. Repeated longitudinal analysis would provide further information regarding sustainability, and widespread validation and safety of this approach could be established in a sufficiently powered, multi-centred future study.

## Data Availability

As study participants did not agree for their data to be shared publicly, supporting data is not publicly available. However, de-identified datasets used and/or analysed for the current study may be available from the corresponding author provided requests are reasonable and do not infringe Victorian health legislation.

## Abbreviations

ED: Emergency department
CT: Computed tomography
CTB: Computed tomography of the brain
CCHR: Canadian CT Head Rule
GCS: Glasgow Coma Scale
LOC: Loss of consciousness
EHHREC: The Eastern Health Human Research and Ethics Committee
VEMD: The Victorian Emergency Minimum Dataset
EMR: Electronic Medical Record
FRAT: Falls Risk Assessment Tool
ICH: Intracranial haemorrhage
DOAC: Direct oral anticoagulant
DAPT: Dual antiplatelet therapy
LMWH: Low molecular weight heparin
